# Behavioral inflexibility in fragile X syndrome: Accounts from caregivers and self-advocates

**DOI:** 10.3389/fpsyg.2023.1118652

**Published:** 2023-02-16

**Authors:** Angelina Jones, Sungeun Kang, Rebecca C. Shaffer, Craig A. Erickson, Lauren M. Schmitt

**Affiliations:** ^1^Department of Pediatrics, Cincinnati Children’s Hospital Medical Center, Cincinnati, OH, United States; ^2^Department of Pediatrics, University of Cincinnati College of Medicine, Cincinnati, OH, United States; ^3^Department of Psychiatry and Behavioral Neuroscience, Cincinnati Children’s Hospital Medical Center, Cincinnati, OH, United States

**Keywords:** behavioral inflexibility, behavioral rigidity, fragile X syndrome, perseveration, repetitive behavior

## Abstract

**Introduction:**

Behavioral difficulties in individuals with fragile X Syndrome (FXS) are one of the primary reasons families seek medical and psychological support. Among these, behavioral inflexibility is very common, and when left untreated, can negatively impact quality of life for the individuals with FXS and their families. Behavioral inflexibility refers to the difficulty in changing one’s behaviors based on environmental demands or social contexts, thus impeding daily functioning, opportunities for learning, and social interactions. In addition to the individual and family impact, behavioral inflexibility is often recognized as a defining phenotype of FXS and appears to be specific to FXS when compared to other genetic forms of intellectual disability. Despite the pervasiveness and severity of behavioral inflexibility in FXS, there are limited measures that adequately assess behavioral inflexibility in FXS.

**Methods:**

We conducted semi-structured virtual focus groups with 22 caregivers, 3 self-advocates, and 1 professional to gather key stakeholders’ perspectives on and experiences of inflexible behavior observed in FXS. Audio-recordings from focus groups were transcribed using NVivo, then verified and coded. Two trained professionals reviewed codes to extract primary themes.

**Results:**

Six themes were extracted: (1) Intolerance of change, (2) Intolerance to uncertainty, (3) Repetitive interests and behaviors, (4) Family impact, (5) Change in behavior across the lifespan, and (6) Impact of the COVID pandemic. Our findings show common examples of these themes included intolerance to disruption to routine, perseverative questioning, watching the same things over and over, and caregivers having to extensively pre-plan for events.

**Discussion:**

The purpose of the current study was to gain key stakeholders’ perspectives *via* focus groups to elicit information and understand patterns of inflexible behaviors in FXS, with the goal of developing a disorder-specific measure to accurately assess behavioral inflexibility across the lifespan and in response to treatment. We were able to capture several phenotypic examples of behavioral inflexibility in FXS as well as their impact on individuals with FXS and their families. The wealth of information gained through our study will aid in our next steps of item generation for measure development of Ratings of Inflexibility in Genetic Disorders associated with Intellectual Disability – Fragile X Syndrome (RIGID-FX).

## 1. Introduction

Fragile X Syndrome (FXS) is the most common inherited form of developmental disability and most common single gene form of autism spectrum disorder (ASD). In FXS, a CGG triplet repeat expansion in the promoter region of the fragile X messenger ribonucleoprotein (*FMR1*) gene (>200 repeats) results in gene methylation and subsequent reduction or absence of production of fragile X messenger ribonucleoprotein (FMRP). Individuals with FXS and their families commonly seek medical and psychological support for behavioral difficulties (Weber et al., 2019), including aggression, self-injury, hyperactivity/impulsivity, and non-compliance (Hagerman et al., 2009). In addition, behavioral inflexibility or rigidity is often clinically observed in individuals with FXS across the lifespan. Behavioral inflexibility refers to the inability or difficulty in changing one’s behaviors based on environmental demands or social contexts. It often can be described as being “stuck” in a repetitive loop of behaviors, which impedes daily functioning, opportunities for learning, and social interactions. Left untreated, behavioral inflexibility can negatively impact quality of life for the individuals with FXS and their families (Bailey Jr et al., 2012). Rigidity and anxiety can have overlapping behavioral manifestations, including repetitive motor behavior and perseverative language (Lozano et al., 2022), often making it hard to distinguish from one another in FXS. Thus, it is crucial to develop a valid and reliable measurement of behavioral inflexibility in FXS that not only assesses the specific behaviors related to rigidity present in individuals with FXS and their severity, but also the degree to which these behaviors interferes with the patients’ and their families’ daily lives. Ultimately, the development of such a measure will be a useful tool to track behavioral flexibility over time and in response to treatment.

In 2009, the National Institutes of Health (NIH) held two meetings of prominent research and practitioners in the FXS field to discuss the advancement of research and targeted treatment, resulting in foundation of the Outcome Measures Working Group. A critical finding recognized by this group was that there were few validated measures specific to FXS (Budimirovic et al., 2017). Currently, there is a lack of adequate FXS-specific assessment measures of inflexible behavior. For example, most available measures are tailored to assess inflexible behavior in autistic individuals, which often differs from symptoms observed in those with FXS (Reisinger et al., 2020). Specifically, the Repetitive Behavior Scale-Revised (RBS-R; Bodfish et al., 1999) and the Flexibility Scale (Strang et al., 2017) exclusively assess inflexible behavior, but were constructed and validated for use in autism, and thus contain items which may not be applicable or appropriate for individuals with FXS. Other measures such as the Aberrant Behavior Checklist (ABC), Anxiety Depression and Mood Scale (ADAMS), and Social Responsiveness Scale (SRS) (Aman et al., 1985; Esbensen et al., 2003; Constantino and Gruber, 2005) assess behavioral challenges more broadly, and thus do not contain enough items relevant to inflexible behaviors.

Of note, none of aforementioned measures assess how inflexible behaviors interfere with daily life, and require caregivers only to rate the severity of behaviors using Likert-like scales. This can impede our understanding of the impact of these behaviors on daily life of individuals and their families as well as how to best treat and detect change in inflexible behaviors. Bodfish et al. (2022) recently developed the Behavioral Inflexibility Scale: Clinical Interview (BIS-CI) to help address this limitation, but this measure was developed for use in ASD. In addition, performance-based outcome measures including the Test of Attentional Performance for Children (Testsysteme, 2011) KiTap Flexibility and NIH-Toolbox Cognitive Battery (NIH-TCB) Dimensions Card Sorting which are often used with individuals with FXS provide objective, quantitative data on behavioral inflexibility, but have limitations of their own. For example, they are often not feasible for individuals with a mental age below 4 years old (Knox et al., 2012; Hessl et al., 2016) and do not necessarily capture true functional impairment and impact on daily life.

Establishing content validity for a new measure is critical in the development process as it ensures that the measurement items adequately reflect the perspective of the target population (Brod et al., 2009). A common method to ensure content validity is to collect qualitative data, which allows researchers to collect experiences and perspectives from individuals with direct interactions with the targeted population. Focus groups serve as a tool to obtain these firsthand perspectives and produce meaningful data on topics with limited research (O'Brien, 1993; Vogt et al., 2004). Subsequently, the information obtained from focus groups can be used for item generation, refinement, and development (Nassar-McMillan et al., 2010). The FXS field recently has made a great effort in prioritizing key stakeholders’ perspectives (i.e., caregivers and self-advocates) (Weber et al., 2019; Lozano et al., 2022; Maertens et al., 2022). Yet, utilizing stakeholder’s perspectives for the measurement development process has been limited in the FXS field, and even in the broader neurodevelopmental and psychiatric community. Thus, the aim of this study is to employ focus groups to gather important stakeholder experiences on behavioral flexibility in FXS with the longer-term goal of creating a caregiver−/self-report measure that is comprehensive, applicable across the lifespan, and sensitive to treatment-related change.

## 2. Materials and methods

Focus groups or 1-on-1 interview sessions were conducted at Cincinnati Children’s Hospital Medical Center (CCHMC) over the course of 8 months to gather information on specific examples of inflexible and rigid behaviors observed in FXS. All focus groups and 1-on-1 sessions took place during the COVID-19 pandemic, and thus occurred *via* secured videoconferencing.

### 2.1. Participants

Twenty-two caregivers (95% female), three self-advocates (100% female), and one professional (100% female) were recruited from the MyFXSResearch portal through the National Fragile X Foundation (NFXF) and CCHMC. All potential participants filled out a survey attached to the focus group advertisement on the NFXF website and were screened by telephone to confirm ≥18 years old and they were English-speaking. Potential caregivers and self-advocates also verbally confirmed that they had at least one child or they themselves had received genetic testing confirming FXS. Caregivers indicated the age-range of their child(ren) with FXS (infancy/toddler: 0–5 years, school-age: 6–12 years, adolescents: 13–17 years, or adult: 18 years and older). One caregivers of an infant/toddler, 12 of school-age children, 2 of adolescents, and 9 of adults participated. All participants provided electronic written consent to the study *via* REDCap. The research qualified as no risk or minimal risk to subjects and was exempt from IRB review, and thus did not collect additional demographic information.

### 2.2. Procedures

#### 2.2.1. Focus group design

The focus groups were moderated by one of two licensed psychologists (LS and RS). We concluded focus groups until thematic saturation was reached. A total of three semi-structured focus groups of caregivers, self-advocates, and professionals were conducted. In addition, due to scheduling conflicts, five participants participated in one-on-one interviews with the primary investigator (LS).

Focus groups and 1-on-1 sessions were used to elicit the perspectives from participants about what behavioral inflexibility looked like in FXS and how it impacted the family. We intentionally recruited caregivers of children with FXS from different age groups in order to best capture rigid behaviors across the lifespan.

#### 2.2.2. Focus group moderator guide

A focus group moderator guide was developed to provide context and structure for the discussions. The Principal Investigator (PI: Schmitt) created a semi-structured guide with examples of inflexible behaviors to help elicit discussions from caregivers based on items from available measures (e.g., RBS-R, Flexibility Scale) and literature regarding inflexible behavior in FXS and more broadly within NDDs (Bodfish et al., 1999; Hagerman et al., 2009; Oakes et al., 2016). Example prompts include “Tell me about any routines or schedules your child has to follow” and “Tell me what happens when there is an unexpected change in your child’s schedule.” In addition, participants were asked to describe how their or their child’s inflexible behaviors impacted their daily lives. Example prompts include “If there is a known change in routine, what do you do to prepare your child?” and “Tell me about activities or situations your family may avoid.”

#### 2.2.3. Data collection

At the start of each group, the moderator reviewed the purpose of the study and reminded participants to respect each other’s confidentially. Next, brief introductions with name, participant type, and if applicable, age-range(s) of child(ren) with FXS were completed. Then, focus group leaders began asking questions from the moderator guide and added follow-up questions when necessary. Each session was audio recorded and a research coordinator took notes to later provide context for the audio-recorded transcripts if needed. The notes served as a backup document to clarify examples participants mentioned and in case an audio recording had technical difficulties. Leaders ensured they were able to develop rapport with the respondents so that participants felt comfortable discussing the sensitive natures of the topics (Fielding and Thomas, 2008). In addition, the focus group leaders did their best to avoid leading and/or insensitive questions.

#### 2.2.4. Audio recording transcription and coding

A post-doctoral fellow and research coordinator transcribed verbatim, verified, and coded each of the nine audio recordings independently using NVivo software (Ltd, 2020). First, the two coders removed any identifying information participants provided and corrected auto-transcribed data by listening to the audio recordings and changing text when necessary. Next, each coder independently reviewed the transcripts to identify and define keywords and phrases. Subsequently, the two coders then agreed on initial codes and went back to the transcripts to verify and create additional codes if needed.

#### 2.2.5. Thematic analysis

Once high internal consistency and validity was established through initial coding, the two psychologists independently reviewed the transcripts and codes to extract themes. Each psychologist generated their own themes and subthemes based on the code. Psychologists initially reached >85% agreement across initial themes and subthemes, and differences were reconciled differences through discussion and additional review of the transcripts.

## 3. Results

Overall, caregivers and self-advocates reported similar concerns related to behavioral inflexibility based on environmental and social demands. Six primary themes were extracted from transcripts: (1) Intolerance to change, (2) Intolerance to uncertainty, (3) Repetitive interests and behaviors, (4) Family impact, (5) Change in behavior across the lifespan, and (6) Impact of the COVID pandemic. For each theme, a series of sub-themes also were identified. In addition, we translated themes (and subthemes) into potential items for the new measure (Table 1).

**Table 1:** Themes included in measurement development.

Theme	No. of coded text segments	Subtheme	Translation into item	Text example
Intolerance of change	23	Routine-oriented	Has difficulty with changes in routine/schedule	*“Like morning time, bedtime, regardless if it’s summer, school, on vacation, whatever, it is the same routine every single day.”*	7	Rule following	Gets upset when others do not follow the rules	*“At night she likes to have the tablets plugged in so it does not happen in the morning. If she wakes up and the tablets aren’t plugged in, then there’s going to be somebody who is going to heard about it, mainly me.”*
5	Difficulty with new places/people	Visiting new places/meeting new people is hard for him/her	*“I just wanted to highlight that (child’s name) had a sub yesterday in one of his general ed[ucation] classes and that just threw them off. And the kid stayed off the whole day.”*
Intolerance of uncertainty	21	Behavior responses to unexpected changes	Has tantrum or outburst (e.g., crying, screaming, stomping) as a result of unexpected change	*“He usually just screams, and his face turns red and he cries like has a total meltdown.”*	24	Perseverative questioning/Asking for details	Repetitively asks questions/seeks out answers about upcoming event even if they were already told/know the answer	*“What are we doing? Who is coming? What are they wearing?”*
Repetitive interests and behaviors	19	Repetitive watching of the same videos, clips, and shows	Watches or listens to same clip/movie/song over and over again	*“Listens to the same two or three songs over and over again.”*
Family impact	19	Planning ahead, back up plans and planning around	My family has to plan in advance of every activity and come up with contingency plans in case he/she has to go home early	*“We always took two cars because we already knew one of us was going home with (child’s name).”*	14	Parent and siblings distress	I worry the impact he/she has on the rest of our family	*“I feel sorry especially for my three daughters, because I know that they sacrificed so much. The whole family does. And I try to do special things with them.”*
3	Avoiding activities/places	My family limits community outings to avoid disruption in his/her life	*“I seem to not be able to handle the people because he can freak out and then embarrass me.”*
8	Visual schedules	Visuals, like social stories, are helpful for my son/daughter in new situations	*“We show him social stories and I show him a video of where we are going and pictures of where we are going.”*

### 3.1. Intolerance to change

Caregivers described behaviors related to how their son or daughter showed intolerance to change in their daily lives. The most common topics highlighted were routine-oriented, rule following, and difficulty with new places and people. For the purposes of this paper, we will use the term “children” to describe the offspring of caregivers who participated in the focus group regardless of the age of the offspring.

#### 3.1.1. Routine-oriented

Caregivers described their children as needing routines in their daily lives. Children had developed certain routines that had to be followed perfectly, especially in the morning and at bed times. Although caregivers spoke of the overall benefit routines provided for their children, they also indicated that if their children’s daily routines were disrupted or changed, they would be need to be made aware of changes in advance and/or would become distressed with the changes.

#### 3.1.2. Rule following

Caregivers reported their children had specific rules for situations, such as only charging their tablets at a certain time of the day or only eating specific foods at certain meals. If these rules were not followed, caregivers explained their child would become upset and insist on the rules to be followed. Although rule following in many of these instances was noted to negatively impact daily functioning, at least one caregiver believed this has helped their child excel in a school setting by following the rules of the classroom.

#### 3.1.3. New places and people

Most caregivers reported their children having difficulty adjusting to new places and people. This difficulty was noted to extend beyond sensory sensitivity concerns. Children often took a while to become comfortable in a new place or situation. In addition, caregivers noted that new situations could negatively impact their child and it would be difficult for their child to recover for the rest of the day. For example, one caregiver reported that their child had a substitute teacher at school without warning, and their child was “off the whole day.” Importantly, caregivers noted that that planning ahead or using visuals prior to a change in people and places occurring was very helpful at reducing anxiety and increasing engagement and comfort in the new situation.

### 3.2. Intolerance to uncertainty

Caregivers repeatedly expressed that their children became upset when they did not know what to expect or did not have all the details about an upcoming event. Two specific behaviors were discussed: perseverative questioning and negative behavioral/emotional responses to unexpected situations.

#### 3.2.1. Perseverative questioning

Nearly all caregivers noted that their children repetitively asked questions Perseverative questioning occurred in two contexts. First, children asked questions about their everyday routines. For example, “What’s for breakfast/lunch/dinner?” and “Where is dad/mom?.” Second, children asked about an upcoming event or change, needing specific and thorough details. For example, “What are we doing? And “Who is coming?.” Perseverative questioning typically occurred throughout the day every day, and occurred even when the children already knew the answer. Due to the level of intensity and frequency of perseverative questioning, many caregivers noted that they often choose not to tell their children about upcoming events too far in advance in order to reduce anxiety and repetitive questions.

#### 3.2.2. Behavior responses to unexpected situations

Caregivers reported a variety of behavioral responses their children demonstrated as a result of unexpected situations, including meltdowns (i.e., screaming, crying), verbal aggression (i.e., saying mean things), and physical aggression (i.e., hitting self or others). Many caregivers were aware of the specific triggers, and thus planned ahead to help minimize the intensity and/or duration of the outbursts. Caregivers also noted that their children often exhibited these behaviors in order to avoid activities outside of their routine (i.e., going to the doctor’s office), and thus over time caregivers expressed that they learned just to “push through” the behaviors while still completing the important events.

### 3.3. Repetitive interests and behaviors

#### 3.3.1. Repetitive watching (or listening) of the same videos, clips, and shows

All caregivers described instances in which their child watched or listened to the same videos and/or shows repetitively. Caregivers most commonly described their children re-watching the same 30 s of a clip. In addition, children watched the same show or movie again and again, even when other family members did not want to. Most caregivers reported this behavior seemed to be a coping mechanism for their child, but others mentioned that repetitively watching the same clip often at the opposite effect and in fact overstimulated their child. For example, one mother said “most of the time it gets to the point of very hyper-arousal, and he just gets overstimulated with it” which resulted in her child yelling at the video. In order to avoid overstimulation, several parents indicated they would give their children options on what to watch, so it was not the same thing every day.

### 3.4. Family impact

Nearly all caregivers shared they frequently needed to adjust their plans and daily lives in order to help their children with FXS manage behavior related to inflexibility. Specifically, caregivers reported planning ahead for changes in routines, using visual aids to support transitions, and avoiding certain activities and places. Of note, caregivers frequently commented on the level of disruption and distress they and other family members experienced as a result of the efforts to minimize their children’s distress related to inflexibility. On the other hand, caregivers also shared how helpful the support strategies they implemented were for their children.

#### 3.4.1. Planning ahead and backup plans

Most families expressed the need to plan ahead, have back up plans, and plan around events since they expect their child to react negatively to a new situation. Many families shared that they use visual aids, including visual schedules or social stories to prepare their children for a new place they will be going and the people they will meet. A few caregivers also mentioned taking their child to the place before an event a couple of times so that the child can become familiar with the environment. In addition, caregivers described developing backup plans in case their children become overly distressed in the new situation. One caregiver said, “We always took two cars because we already knew one of us was going home with (child’s name),” and nearly all caregivers in the same focus group agreed with this statement. Although backup plans and planning around events for the child is extra work for the caregiver, all the caregivers noted the immense benefit it has on ensuring their child does not become overly distressed.

#### 3.4.2. Avoiding activities and places

Several caregivers reported avoiding or limiting community outings to avoid disrupting their child’s routine and upsetting them. Although preparing ahead and showing visual aids before an event can be helpful, a few caregivers noted that they often choose to not try new things or even avoid a known situation if they suspect their child would react negatively. Although avoidance of activities and places was often to minimize the child’s distress, several caregivers shared they avoided situations because of the negative impact on themselves. For example, one caregiver reported, “I seem to not be able to handle the people because he can freak out and then embarrass me.”

#### 3.4.3. Parent and siblings’ distress

While planning ahead and creating backup plans for new situations is beneficial for the child with FXS, many caregivers reported feeling overwhelmed and tired by how much goes into the planning. Some often feel anxious themselves thinking about how their child with react to the change and how unpredictable it can be. An important point that came up was the impact of this on the siblings of those with FXS. Many families believe the non-FXS siblings often miss out on experiences in their lives to make the child with FXS more comfortable. Caregivers reported worrying about the impact on the rest of the family and consciously trying to do special activities with them so they knew they were not forgotten.

### 3.5. Themes not included in measurement development

The following two themes were identified during the focus groups, though these themes will not specifically be translated into items for the measurement. However, given the importance of change across the lifespan, it is our hope that the measure and its items, in general, will be applicable across the lifespan and be able to track over time. Given all focus groups and interviews were conducted during the COVID-19 pandemic, it is not surprising that this emerged as a theme. The majority of behavioral examples noted within this theme also were noted in other themes, and thus will be more broadly translated into items.

#### 3.5.1. Change across the lifespan

Nearly all caregivers of older children with FXS that participated in the focus groups reported a decreased severity and/or frequency of inflexible behaviors in adolescence and adulthood compared to the behaviors they observed when their children were younger. These caregivers specifically reported a decrease in preservative speech and repetitive behaviors with age. Older individuals with FXS also were noted to handle transitions between activities better than their younger selves. One caregiver reported their child did not seem to need their support as much. Although certain behaviors related to inflexibility decreased over time, many caregivers still reported persistence of difficulties in two areas: anxiety with new situations and requiring details prior to new activities.

#### 3.5.2. COVID impact

Many caregivers expressed how the need to “lock down” due to the COVID-19 pandemic impacted their children negatively. They mentioned how transitioning between places and people was better pre-pandemic compared to current functioning when most things were closed or virtual. With the disruptions to their routine, children often struggled with the adjustment to online school and were not able to attend their usual extracurricular activities. At the time of the focus groups, some children were in the process of returning to school, and were struggling to go back to their former routines since they became used to the “pandemic way” of doing things. In addition, new concerns regarding safety of leaving the house were reported. One caregiver stated, “We have learned to stay home and feel safe at home, so that definitely impacts us going outside and to community activities.”

## 4. Discussion

Utilizing focus groups to gather qualitative information from key stakeholders, we offer important new insights into how behavioral inflexibility manifests in FXS and its impact on the daily lives of individuals with FXS and their families. Our findings indicate the pervasiveness of behavioral inflexibility across the lifespan and settings (i.e., home, school, work, community) in FXS. We identified six themes from our focus groups: (1) Intolerance to change, (2) Intolerance to uncertainty, (3) Repetitive interests and behaviors, (4) Family impact, (5) Change in behavior across the lifespan, and (6) Impact of the COVID pandemic. Within themes and subthemes, caregivers often reported nearly identical examples of manifestations of behavioral inflexibility (e.g., strict routines around bedtime, perseverative questioning prior to an event, watching the same thing over and over, and caregivers having to extensively pre-plan for events), suggesting certain behaviors may be more specific to FXS rather than more broadly across neurodevelopmental disorders. In addition, nearly all families expressed the need for the family to be flexible when their child could not be (e.g., planning head, having backup plans). Taken together, in the first study of its kind in FXS, our focus groups were able to elucidate the impact of inflexible behaviors on families, including the extent to which caregivers accommodate inflexible behaviors in their daily family life. This is an important contribution to the field as it not only incorporates key stakeholders’ perspectives on how behavioral flexibility manifests in FXS, but also provides a rich sample of information from which a new measure of behavioral inflexibility can be generated.

Our findings closely mirror what has been reported in medical and psychological settings in regards to behavioral difficulties in FXS, with caregivers highlighting how these behaviors can negatively impact their child’s quality of life (Hagerman et al., 2009; Weber et al., 2019). Additionally, studies indicate over 80% of individuals with FXS demonstrate behavioral inflexibility (Abbeduto et al., 2007; Moss et al., 2009; Oakes et al., 2016) in particular verbal perseveration which is often recognized as one of the defining phenotypes of FXS (Abbeduto et al., 2007; Roberts et al., 2008; Moss et al., 2009). Our study further highlights the prevalence of verbal perseveration and repetitive speech and how much it can impede daily functioning in these individuals and their families. Interestingly, one caregiver reported their child’s need for routine and rule-following can actually benefit them, specifically in a school setting. This importantly suggests that behaviors often viewed as inflexible or rigid can be beneficial and functional in certain situations.

Importantly, caregivers reported the impact of inflexible behaviors on their families, which is a current limitation of available measures. Although Bodfish et al. (2022) recently addressed this limitation in ASD, given our focus group findings suggest behavioral inflexibility may manifest uniquely in FXS, it is important to future measures specific to FXS assess both the individual and family impact. Specifically, many caregivers reported avoiding or limiting community outings to avoid disrupting their child’s routine and often felt overwhelmed and tired by how much energy they devote to planning. It is important to capture how behavioral inflexibility in children with FXS impacts caregivers’ relationships with others, their ability to complete important tasks (e.g., doctor’s appointments), and the level of support, time, and effort their children need to be successful in daily life.

### 4.1. Clinical implications and recommendations

Our findings provide a comprehensive overview of inflexible behaviors in individuals with FXS, which have important clinical and research implications. Given the prevalence and severity of inflexibility in FXS, it is important that care providers (i.e., physicians, clinical psychologists, speech pathologists, etc.) speak with families about the presence of these specific behaviors in this patient population. It also is important to assess whether inflexibility co-occurs with anxiety or whether inflexibility is occurring independently. Our focus group thematic data provides a useful guide that providers may use during clinical interviews to probe specific behavioral manifestations of inflexibility in FXS. As such, we recommend providers and families consider treatment targets of specific inflexible behaviors, especially those that cause the individual with FXS and/or their families high levels of distress. Relatedly, we recommend providers assess the level of impact these behaviors have on caregivers as they may have their own support needs. In addition, we recommend families and their education team work together to develop behavior plans and/or specific individualized education plan (IEP) goals if inflexible behaviors are interfering at school. Our focus groups highlight common behavioral strategies that are helpful in minimizing the negative impact of changes in routine, including using visual supports and developing back-ups plans. Thus, providers, school personnel, and families are encouraged to implement strategies identified as helpful by caregivers of individuals with FXS. Although inflexible behavior is highly prevalent and often functionally impairing in FXS, our focus groups also offer hope to families since the majority of caregivers of adults with FXS reported that inflexible behaviors lessen in severity and impact over time and even in some cases disappear in adulthood. Last, in the area of research, we recommend future studies focus on better understanding the pathophysiological mechanisms underlying inflexible behavior as well as determine the extent to which rigidity and anxiety have distinct versus overlapping biological mechanisms in FXS. These studies will be critical to speeding potential pharmacological treatment that may be used in combination with behavioral intervention approaches.

### 4.2. Limitations

Due to the COVID-19 pandemic, we were limited to conducting focus groups and 1-on-1 sessions *via* a virtual format. There also were time constraints on when caregivers could participate due to an increase in caretaking responsibilities with their children home full time, resulting in a reduction in their ability to participate in group sessions. Using focus groups is ideal for obtaining meaningful data on specific topics and obtaining diverse perspectives. Although everyone was encouraged to participate, we observed some group members shared more than others, which could have possibly influenced the overall group discussion. Additionally, though we recruited caregivers of individuals with FXS from infancy to adulthood, we had an uneven distribution of caregivers across different stages of development. Since we reached data saturation within a small number of sessions, these remain only minor limitations. Last, we had very limited demographic information due to the study being IRB exempt. Knowing the participant’s background information would have been beneficial for readers to understand how different socio-economic statuses, languages spoken at home, and race/ethnicity differ between the families and impact their child’s accessibility to resources to help with inflexible behaviors. In addition, we had few self-advocates participate, and males were under-represented across participant groups perhaps due to the language-based nature of the focus group format. Thus, our findings may be limited to perspectives of mothers of individuals with FXS.

## 5. Conclusion

Our focus groups conducted with caregivers of individuals with FXS and self-advocates provide further insights into how behavioral inflexibility manifests in FXS and its overall impact in daily life in this patient population. Our findings provide examples of behavioral inflexibility in FXS (i.e., perseverative questioning), suggesting certain behaviors may be more specific to FXS rather than broader neurodevelopmental disorders, an area that has been relatively under-studied in this field. Together, our findings highlight an important area of continued clinical and research focus as well as provide are a critical step to developing the first measure of inflexible behavior for individuals with FXS, Ratings of Inflexibility in Genetic Disorders associated with Intellectual Disability – Fragile X Syndrome (RIGID-FX) that can be used in research and clinical practices across the lifespan.

## Data availability statement

The original contributions presented in the study are included in the article/supplementary material, further inquiries can be directed to the corresponding author/s.

## Ethics statement

The studies involving human participants were reviewed and approved by Cincinnati Children’s Hospital Medical Center. The patients/participants provided their written informed consent to participate in this study.

## Author contributions

LS contributed to conception and design of study. AJ and LS organized the database. AJ, SK, LS, and RS performed the data analysis. AJ wrote the first draft of the manuscript. LS, SK, RS, and CE contributed to manuscript revision. All authors approved the submitted version.

## Funding

This study was supported by the Jack H. Rubinstein Foundation.

## Conflict of interest

The authors declare that the research was conducted in the absence of any commercial or financial relationships that could be construed as a potential conflict of interest.

## Publisher’s note

All claims expressed in this article are solely those of the authors and do not necessarily represent those of their affiliated organizations, or those of the publisher, the editors and the reviewers. Any product that may be evaluated in this article, or claim that may be made by its manufacturer, is not guaranteed or endorsed by the publisher.
